# Endothelial chemokine receptors as facilitators of tumor cell extravasation?

**DOI:** 10.18632/oncotarget.672

**Published:** 2012-09-19

**Authors:** Alexandra Hoos, Monika J. Wolf, Judith Bauer, Lubor Borsig, Mathias Heikenwalder

**Affiliations:** Institute of Physiology, Zurich Center for Integrative Human Physiology, University of Zurich, Zurich, Switzerland; Institute of Surgical Pathology, University Hospital Zurich, Zurich, Switzerland; Institute of Virology, Technische Universität München/Helmholtz Zentrum Munich, Munich, Germany; Institute of Physiology, Zurich Center for Integrative Human Physiology, University of Zurich, Zurich, Switzerland; Institute of Virology, Technische Universität München/Helmholtz Zentrum Munich, Munich, Germany

Metastasis is a multistep process characterized by the ability of tumor cells to “communicate” and to interact with their microenvironment to establish tumors in distant organs. A significant proportion of the metastatic microenvironment consists of leukocytes, mostly of the innate immune system, contributing to tumor invasion and outgrowth. Chemokines are instrumental for recruiting immune cells thereby enabling efficient metastasis [1]. Still, the exact mechanisms of chemokine action in the metastatic process have remained elusive. In the context of cancer, chemokines were shown to affect leukocyte recruitment, tumor cell proliferation, invasion, angiogenesis and cancer progression [2]. In addition, tissue-specific metastasis of certain cancers was shown to depend on specific chemokine-chemokine receptor interactions. For instance, the expression of the chemokine receptor CXCR4 on breast cancer cells supports metastasis to secondary sites expressing CXCL12 [3]. Chemokine-driven leukocyte recruitment in tumors is linked to the major attractors of monocytic cells, namely CCL2 and CCL5 [4, 5]. Elevated levels of CCL2 correlated with poor prognosis due to metastasis in a variety of cancer patients e.g. suffering from breast and prostate cancer. In a recent study we detected elevated levels of CCL2 in biopsies from metastatic colon cancer patients (UICC stage IV) [6].

Still, the different roles of CCL2 and CCL5 during metastasis are just at the beginning to be defined. In the tumor microenvironment, stromal cells, infiltrating leukocytes and tumor cells themselves were identified as sources of chemokines. During the initial phase of metastasis, local activation of endothelia by tumor cells induced endothelial CCL5 expression, resulting in monocyte recruitment and promotion of metastasis [7]. Recently, CCL2-mediated recruitment of monocytes has been identified as the major factor facilitating breast cancer metastasis to the lung [8], while the exact role of monocytes in this process remained unanswered. We showed that monocytes enable tumor cell transmigration through the endothelium [6]. Using mouse models we demonstrated that expression of CCL2 by colon cancer cells not only attracts pro-inflammatory monocytes but also directly signals towards CCR2+ endothelial cells to enable tumor cell extravasation. Importantly, tumor cells were unable to efficiently transmigrate through CCR2-deficient endothelial cells, indicating the presence of CCR2 on the endothelium is a crucial factor for CCL2-mediated metastasis. Activation of CCR2 on endothelial cells through the JAK2-Stat5 and p38MAPK pathway caused increased vascular permeability and enhanced tumor cell transmigration *in vivo* and *in vitro*. Moreover, blocking of signaling pathways downstream of CCR2 significantly reduced metastasis, supporting the notion that endothelial CCR2 “licenses” colon cancer extravasation. Consequently, our findings define an additional role for chemokines in enabling metastasis that goes beyond the attraction of inflammatory leukocytes.

We confirmed that metastasis of another tumor cell line, Lewis lung carcinoma (3LL), is also dependent on CCL2 expression. In contrast, the melanoma cell line B16-BL6 could extravasate and metastasize independently of endothelial CCR2 expression. Interestingly, this cell line expresses only little CCL2. Our findings raise several questions:

(1) Do other cancer cells also use chemokine-chemokine receptor interactions to accomplish tumor cell extravasation?
(2) Can we use chemokine expression analysis in primary tumors of patients to predict increase risk for metastasis?
(3) What other chemokine-chemokine receptor pairs exist, having the same or other biological function as CCL2-CCR2?
(4) What do we know about the cellular and spatiotemporal denominators of CCL2-induced tumor cell extravasation?

Consequently, further experiments will show how general our recently reported findings are and whether other chemokine receptors on endothelial cells exert similar functions. We hypothesize that other functions of tumor cell-derived chemokines will be identified in the future, surpassing their known role as immune cell attractors, as the currently identified contribution to induced vascular permeability.
